# Digital Health Interventions for Delivery of Mental Health Care: Systematic and Comprehensive Meta-Review

**DOI:** 10.2196/35159

**Published:** 2022-05-12

**Authors:** Tristan J Philippe, Naureen Sikder, Anna Jackson, Maya E Koblanski, Eric Liow, Andreas Pilarinos, Krisztina Vasarhelyi

**Affiliations:** 1 Department of Cellular & Physiological Sciences The University of British Columbia Vancouver, BC Canada; 2 Department of Psychiatry The University of British Columbia Vancouver, BC Canada; 3 Vancouver Community College Vancouver, BC Canada; 4 School of Social Work The University of British Columbia Vancouver, BC Canada; 5 Department of Psychology The University of British Columbia Vancouver, BC Canada; 6 Vancouver Coastal Health Research Institute Vancouver, BC Canada; 7 School of Population and Public Health The University of British Columbia Vancouver, BC Canada; 8 Faculty of Health Sciences Simon Fraser University Burnaby, BC Canada

**Keywords:** digital health, telepsychology, computer-assisted therapy, online therapy, mobile applications, mobile apps, telemedicine, telepsychiatry, virtual reality exposure therapy, mental health, COVID-19

## Abstract

**Background:**

The COVID-19 pandemic has shifted mental health care delivery to digital platforms, videoconferencing, and other mobile communications. However, existing reviews of digital health interventions are narrow in scope and focus on a limited number of mental health conditions.

**Objective:**

To address this gap, we conducted a comprehensive systematic meta-review of the literature to assess the state of digital health interventions for the treatment of mental health conditions.

**Methods:**

We searched MEDLINE for secondary literature published between 2010 and 2021 on the use, efficacy, and appropriateness of digital health interventions for the delivery of mental health care.

**Results:**

Of the 3022 records identified, 466 proceeded to full-text review and 304 met the criteria for inclusion in this study. A majority (52%) of research involved the treatment of substance use disorders, 29% focused on mood, anxiety, and traumatic stress disorders, and >5% for each remaining mental health conditions. Synchronous and asynchronous communication, computerized therapy, and cognitive training appear to be effective but require further examination in understudied mental health conditions. Similarly, virtual reality, mobile apps, social media platforms, and web-based forums are novel technologies that have the potential to improve mental health but require higher quality evidence.

**Conclusions:**

Digital health interventions offer promise in the treatment of mental health conditions. In the context of the COVID-19 pandemic, digital health interventions provide a safer alternative to face-to-face treatment. However, further research on the applications of digital interventions in understudied mental health conditions is needed. Additionally, evidence is needed on the effectiveness and appropriateness of digital health tools for patients who are marginalized and may lack access to digital health interventions.

## Introduction

Patients with mental health conditions often experience long-term disability, resulting from challenges in accessing mental health services, including low treatment availability and long wait times [1]. Moreover, the COVID-19 pandemic has exposed crucial gaps in mental health care systems, which significantly impact the well-being of many people globally [2-4]. Increased fears of contracting SARS-CoV-2, the burden of quarantine requirements, social distancing, social isolation, rising economic inequities, unemployment, and new workplace requirements are additional stressors brought on by the pandemic, which can exacerbate the symptoms of mental health conditions [5-14]. The pandemic is thought to account for recent increases in mood, anxiety, trauma, and substance use disorders [10-16]. Similar trends in mental illness were observed during the 2003 severe acute respiratory syndrome outbreak, other previous pandemics [10-12,17,18], and recent economic crises [10-12,17,18]. The rise in mental health issues due to the COVID-19 pandemic creates substantial pressures on an already strained mental health care system [12,19], with evidence pointing to a silent mental health crisis as resources are prioritized for stemming the spread of SARS-CoV-2 infections [12].

Consequently, interest in web-based health service delivery has been growing in recent years. These include synchronous and asynchronous therapist contact via messaging, phone call, and videoconferencing; computer, web-based, and mobile delivery of therapy programs; virtual or augmented reality–based programs; computerized or web-based cognitive training, and web-based peer and social support groups (defined below). The global reach of digital health care potentially extends to billions of people with internet access. Web-based and mobile delivery of therapy programs may save practitioner time owing to efficient and effective delivery of treatments at lower associated cost [20]. Digital health interventions may also offer a way to reduce or avert care interruptions while allowing practitioners to adhere to safe social distancing measures [20]. At the onset of the COVID-19 pandemic, health care providers rapidly transitioned to web-based health care delivery to limit the risk of COVID-19 transmission. However, the state of the evidence on the effectiveness of digital interventions is unclear, and the implications for health outcomes of such a drastic shift to digital health platforms are difficult to predict [12,21-25]. Whether clinicians can provide effective and reliable treatment, perform assessments [26,27], identify ailments and symptoms [28], manage suicidal behaviors [26,28,29], and provide personable, compassionate services [26,30,31] remains uncertain. Furthermore, digital delivery of services may be complicated by the symptomatology of some mental health conditions [26,29], concurrent medical conditions [29], and socioeconomic factors [31-42]. A lack of information, resources, and understanding of complex patient-related factors could negatively affect care delivery and overall patient health.

Mobile apps are increasingly used by the public for the treatment of mood and anxiety disorders, sometimes without professional referral or guidance [13,43,44]. There is also some evidence that web-based forums and resources are increasingly common [45-54]. Similarly, over the past decade, there have been noticeable shifts in the provision of cognitive and behavioral training for developmental disorders and dementia to computer and other web-based platforms [55-64]. There are also significant developments in the application of virtual reality tools in health care settings [65-67]. The need for professional guidance in the use of web-based or mobile services and forums is subject to controversy [68-77], and more evidence is needed on optimal ways to integrate these tools into a comprehensive approach to mental health care.

This review is motivated primarily by questions from health care stakeholders in a Canadian setting, who were required to rapidly shift to digital delivery of mental health services during the COVID-19 pandemic. However, to date, there has been no comprehensive review on the use of digital interventions for the treatment of a representative range of mental health conditions. With the present meta-review, we seek to fill this gap and summarize existing evidence on the use of digital health interventions in mental health care. Our hope is that our review will be used by health care stakeholders to inform their consideration of mental health care options for digital delivery.

## Methods

### Literature Search

We conducted a review of peer-reviewed literature examining the application of digital health interventions for the treatment of mental health conditions described below. We searched Medline on November 1, 2021, for research published after January 1, 2010. We used Medline filters to restrict retrieved records to meta-analyses, systematic reviews, and other types (narrative and conceptual) of literature reviews. We used broad term definitions to maximize the types of digital health interventions and mental health conditions captured in the search. The search strategy consisted of combinations of Medical Subject Headings (MeSH) words and other keywords including the following: virtual reality; telemedicine; computer-assisted therapy; digital health; videoconferencing; mental health; mental health services; psychotherapy; attention deficit and disruptive behavior disorders; anxiety disorders; trauma and stressor related disorders; mood disorders; bipolar and related disorders; dementia; disruptive, impulse control and conduct disorders; dissociative disorders; feeding and eating disorders; neurodevelopmental disorders; neurotic disorders; pain; personality disorders; schizophrenia spectrum and other psychotic disorders; sleep wake disorders; and substance-related disorders (see search query in Multimedia Appendix 1).

### Inclusion and Exclusion Criteria

This review is restricted to other reviews that assessed the use of digital interventions for the treatment of mental health conditions. Studies that did not report on the effectiveness of digital interventions on mental health outcomes or did not outline a study protocol were excluded from this review.

### Data Extraction, Analysis, and Quality Assessment

Once records were retrieved and deduplicated, TJP, NS, and AJ conducted title and abstract screening where any disagreements were resolved through consensus. Team members then proceeded with mutually exclusive full-text screening to identify articles that qualified for inclusion in the review. As with previous meta-reviews, adherence to Preferred Reporting Items for Systematic Reviews and Meta-Analyses (PRISMA) guidelines [78] was considered to assess risk of bias in selected studies (maximum score of 1: completely adheres to PRISMA Guidelines). To assess the quality and reliability of research within the field, one reviewer conducted data extraction using a standardized and iterative data extraction form (Multimedia Appendix 2). Extracted data included study details (author, date, and type), participant characteristics (mental health conditions), intervention details (intervention type and effectiveness), number of participants, and controls used (treatment as usual, waitlist, placebo, or not applicable). Quality and bias scores describing the primary literature reported in included studies were averaged and faithfully converted (when necessary) to a consistent 3-point scale (1=low, 2=moderate, and 3=high). Owing to the significant heterogeneity in research approaches and findings, we selected a qualitative and semiquantitative approach to summarize and present research findings.

### Organization of Mental Health Conditions

To provide a clearer picture of how digital interventions are used in treating various mental health conditions, we separated mental health conditions based on the parent MeSH terms and Diagnostic and Statistical Manual of Mental Disorders, Fifth Edition (DSM-5) criteria. Where we identified dissimilarities or similarities in treatment, we either added a subcategory or combined categories together. We removed attention-deficit/hyperactivity disorder (ADHD) from developmental disorders and added it as a separate category owing to dissimilarities in the treatment and management of this condition versus other developmental disorders. We combined the frequently comorbid anxiety, mood, and trauma disorders owing to similar treatment approaches, effectiveness, and reporting in the literature. Additionally, patients with chronic pain, chronic medical illnesses, and chronic disabilities (shortened to chronic illnesses) often experience mental health issues that are underrecognized, receive little attention within digital health intervention literature [79-83], and have unreliable treatment efficacies [79-83]. Despite the use of similar psychological treatments anxiety, mood, and trauma disorders [84-87], chronic illness treatments also involve acceptance, remediation, music, and virtual reality [79-82,84-86]. We therefore retain chronic illness as a category related to but separate from anxiety, mood, and trauma disorders. Similarly, caregivers are often untrained family members who face significant stress and anxiety in the process of providing care for loved ones. Caregivers also benefit from mental health services such as cognitive behavioral therapy and specific psychoeducation, which overlap with some mental health conditions. but also benefit from peer support, training, and acceptance therapy [70,88-95]. Substance use disorder was included since treatments include therapies based on psychological principles [32,78,96-115], and this disorder is often comorbid with other mental health disorders and is considered a mental health condition by medical associations (eg, Canadian Medical Association, American Medical Association, and World Health Organization) and diagnostic manuals (eg, DSM-5).

This review adheres to PRISMA guidelines [116] (Multimedia Appendix 1).

## Results

### Included Studies

**Figure 1:**
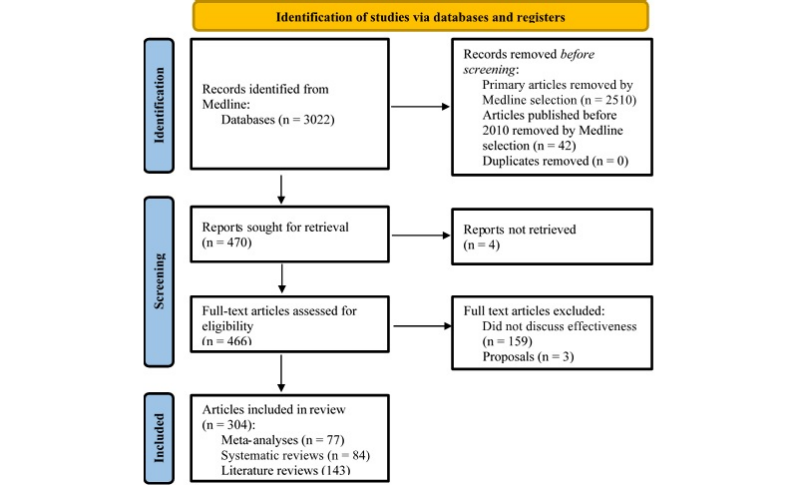
PRISMA (Preferred Reporting Items for Systematic Reviews and Meta-Analyses) flowchart for study selection.

The PRISMA flowchart of the screening process is presented in Figure 1. We identified 3051 records and used Medline selection tools to exclude primary articles (n=2510), and studies published before 2010 (n=42). Of the remainder, 4 were inaccessible and authors did not respond to copy requests; thus, 466 studies proceeded to full-text review where 159 were excluded for not reporting on intervention effectiveness and 3 were proposals. This selection resulted in 77 meta-analyses, 84 systematic reviews, and 143 literature reviews examining the use of digital health interventions for the treatment of mental health conditions.

### Mental Health Conditions

**Figure 2:**
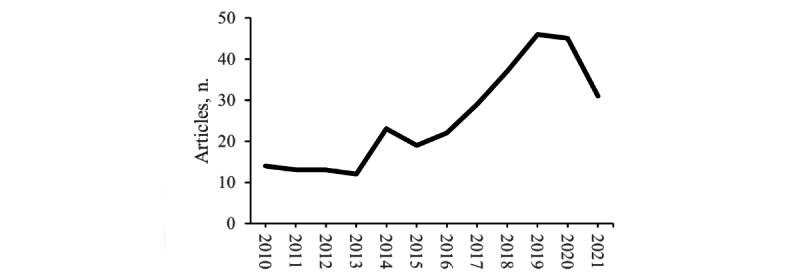
Number of included articles per year.

A summary of metadata extracted from database searches, curated secondary literature, curated primary literature, and participant numbers is provided in Table 1. Per participant, studies on substance use disorders account for a majority (n=241,377, 52%) of digital mental health research, followed by mood, anxiety, and trauma disorders (136,121, n=29%), and >5% for other mental health conditions (pain: n=24,327, schizophrenia: n=20,500, dementia: n=10,823, feeding and eating: n=10,441, developmental: n=8736, bipolar: n=3573, sleep-wake: n=3333; and ADHD: n=2428). Additionally, limited research has examined the use of digital health to provide psychological support to caregivers of people with dementia and developmental disorders. Lastly, we retrieved no records examining the use of digital health interventions to treat antisocial, avoidant, borderline, dependent, histrionic, and narcissistic personality, dissociative identity, paraphilic, and sexual health disorders. To demonstrate how the amount and reliability of research can be estimated from metadata, we correlated elements in Table 1 and report a 4D correlation (*P*<.05; see Table S2 and 4D illustration in Figure S1 in Multimedia Appendix 1). Overall, this illustrates a significant need for the development and testing of digital interventions for other mental health conditions. Nevertheless, the number of research publications has steadily increased since 2015 (Figure 2)—a trend that will likely continue with greater interest in digital mental health research.

**Table 1:** Metadata per mental health condition examining article and participant numbers.

Mental health conditions	Total literature^a^, n	Secondary literature^b^, n	Primary literature^c^, n	Participants, n (%)
Attention-deficit/hyperactivity disorder	90	8	35	2428 (0.5)
Anxiety, mood, stress, trauma	1205	123	923	136,121 (29.5)
Bipolar and related disorders	65	9	42	3573 (0.8)
Dementia	246	24	180	10,823 (2.3)
Developmental disorders (excluding attention-deficit/hyperactivity disorder)	326	24	349	8736 (1.9)
Feeding and eating disorders	154	23	117	10,441 (2.3)
Pain	147	23	348	24,327 (5.3)
Schizophrenia and psychotic disorders	263	30	304	20,500 (4.4)
Sleep-wake disorders	145	8	29	3333 (0.7)
Substance-related disorders	555	59	466	241,377 (52.3)

^a^Total number of articles from Medline searches.

^b^Selected secondary literature.

^c^Primary literature curated by secondary sources.

### Digital Health Interventions

To more precisely measure the amount of research available to treat specific mental health conditions using a specific digital health intervention, we superimposed the primary digital health interventions on study characteristics and conclusions drawn from our search results (Table 2). All digital health interventions are supplementary to synchronous or real-time communication.

**Table 2:** Digital health interventions used to treat mental health conditions.

Condition			Therapist contact		Online peer support		Web-based therapy		Mobile therapy		Virtual reality		Cognitive training
**Attention-deficit/hyperactivity disorder**													
	RCT-TAU^a^ (patients/studies), n/n	45/1		—^b^		—		—		—		363/4	
	RTC-other^c^ (patients/studies), n/n	968/6		—		—		—		—		929/14	
	Observational (patients/studies), n/n	45/4		—		—		—		2/1		36/5	
	Reported study quality (1=low to 3=high)	Not reported		—		—		—		1.00		1.00	
	Overall strength of evidence^d^	Medium		—		—		—		Low		Medium	
	Effective as treatment modality?^e^	Yes		—		—		—		Emerging		Inconclusive	
	Effective as assessment modality?^e^	Yes [55,56,117-119]		—		—		—		Emerging [56,120,121]		Inconclusive [56,57,117,118, 122]	
**Anxiety-, mood-, stress-, and trauma-related disorders**													
	RCT-TAU (patients/studies), n/n	7156/60		—		19,803/105		1333/6		2842/65		42/3	
	RTC-other (patients/studies), n/n	5460/38		73/1		51,074/279		3905/22		2974/69		222/6	
	Observational (patients/studies), n/n	1567/41		—		31,461/93		45/1		305/21		—	
	Reported study quality (1=low to 3=high)	2.33 (SD 0.57)		Not reported		2.38 (SD 0.68)		1.64 (SD 0.64)		1.78 (SD 0.68)		1.00	
	Overall strength of evidence	High		Low		High		Medium		High		Low	
	Effective as treatment modality?	Yes		Emerging		Yes		Yes		Yes		Emerging	
	Effective as assessment modality?	Yes [68,96,123-144]		No studies [129]		Emerging [1,30,31,68, 69,84-86,96, 118,124,125,129, 130,133-135,137, 138,142,144-192]		Yes [1,43,68,96, 144,185-187,191, 193-204]		Emerging [65-67,134,183, 184,205-229]		Emerging [166,230]	
**Bipolar and Related Disorders^f^**													
	RCT-TAU (patients/studies), n/n	—		—		992/14		132/1		—		—	
	RTC-other (patients/studies), n/n	14/1		286/3		1499/7		—		—		—	
	Observational (patients/studies), n/n	—/3		156/1		273/5		51/1		—		—	
	Reported study quality (1=low to 3=high)	Not reported		1.00		1.00		2.75		—		—	
	Overall strength of evidence	Low		Medium		Medium		Low		—		—	
	Effective as treatment modality?	Yes		Emerging		No^j^		Emerging		—		—	
	Effective as assessment modality?	Yes [231,232]		No studies [233]		No studies [231-235]		No studies [201,231,236,237]		[231]		—	
**Dementia and Related Disorders**													
	RCT-TAU (patients/studies), n/n	483/6		—		—		3981/7		331/11		590/16	
	RTC-other (patients/studies), n/n	486/4		—		—		—/30		30/1		1222/19	
	Observational (patients/studies), n/n	1695/29		—		—		—/30		273/10		282/16	
	Reported study quality (1=low to 3=high)	1.75 (SD 0.75)		—		—		Not reported		1.50 (SD 0.50)		1.63 (SD 0.41)	
	Overall strength of evidence	Medium		—		—		Medium		Medium		Medium	
	Effective as treatment modality?	Yes		—		—		Yes		Inconclusive		Yes	
	Effective as assessment modality?	Yes [58,238-245]		—		—		Yes [59,60,240,242,246]		No studies [221,240,242, 247-249]		Yes [61-64,166,240, 242,250-252]	
**Dementia: caregiver support**													
	RCT-TAU (patients/studies), n/n	773/8		11/1		1054/10		—		—		—	
	RTC-other (patients/studies), n/n	1019/10		384/4		2852/17		—		—		—	
	Observational (patients/studies), n/n	78/3		54/2		176/6		—		—		—	
	Reported study quality (1=low to 3=high)	1.50		1.50		1.50		—		—		—	
	Overall strength of evidence	Medium		Low		Medium		—		—		—	
	Effective as treatment modality?	Yes		Emerging		Yes		—		—		—	
	Effective as assessment modality?	Yes [88,89,240]		No studies [70,88-91]		Emerging [70,88,89]		—		—		—	
**Developmental disorders (excluding attention-deficit/hyperactivity disorder)**													
	RCT-TAU (patients/studies), n/n	535/13		—		—		107/4		222/7		984/20	
	RTC-other (patients/studies), n/n	327/10		—		80/3		69/3		877/24		1298/55	
	Observational (patients/studies), n/n	3330/88		—		7/1		7/2		212/24		392/37	
	Reported study quality (1=low to 3=high)	1.56 (SD 0.77)		—		1.00		1.50		2.50 (SD 0.50)		1.00 (SD 0)	
	Overall strength of evidence	High		—		Low		Low		Medium		High	
	Effective as treatment modality?	Yes		—		Inconclusive		Inconclusive		Inconclusive		Yes	
	Effective as assessment modality?	Yes [92,119,253-263]		—		No studies [119,263]		No studies [264]		No studies [120,221,253,265,266]		Yes [118,253,267-270]	
**Feeding and eating disorders**													
	RCT-TAU (patients/studies), n/n	833/15		—		2497/23		276/3		628/9		—	
	RTC-other (patients/studies), n/n	97/1		275/3		3361/29		143/3		—		—	
	Observational (patients/studies), n/n	296/8		—		1928/18		107/5		—		—	
	Reported study quality (1=low to 3=high)	2.00		2.00		1.83 (SD 0.69)		1.00		1.00		—	
	Overall strength of evidence	Medium		Medium		High		Medium		Medium		—	
	Effective as treatment modality?	Yes		Emerging		Yes		Emerging		Yes		—	
	Effective as assessment modality?	Yes [28,71,72,271,272]		No studies [72]		Emerging [71-74,171,272-278]		Emerging [74,276,279-282]		No studies [120,211,283-285]		N/A	
**Chronic pain, disability, and other medical illness**													
	RCT-TAU (patients/studies), n/n	4350/54		—		1339/10		0		3583/43		—	
	RTC-other (patients/studies), n/n	3203/46		—		5666/37		—		2642/35		—	
	Observational (patients/studies), n/n	2066/66		—		—		—		1478/56		—	
	Reported study quality (1=low to 3=high)	1.42 (SD 0.55)		—		2.0 (SD 0.71)		—		1.21 (SD 0.37)		—	
	Overall strength of evidence	High		—		High		—		High		—	
	Effective as treatment modality?	Yes		—		Yes		—		Yes		—	
	Effective as assessment modality?	No studies [286,287]		—		Emerging [79,80,85,286-289]		—		No studies [81-83,290-301]		—	
**Schizophrenia and psychotic disorders^g^**													
	RCT-TAU (patients/studies), n/n	—		—/4		—/9		1580/6		292/4		1495/33	
	RTC-other (patients/studies), n/n	3287/5		—/2		101/3		5837/4		1735/28		1783/31	
	Observational (patients/studies), n/n	404/5		—/2		20/2		1891/23		1267/32		86/5	
	Reported study quality (1=low to 3=high)	1.50 (SD 0.87)		1.67 (SD 0.94)		2.00 (SD 1.00)		1.83 (SD 0.62)		2.25 (SD 1.0)		2.50 (SD 0.41)	
	Overall strength of evidence	Medium		Low		Low		Medium		High		High	
	Effective as treatment modality?	Yes		Yes		Yes		Yes		Inconclusive		Yes	
	Effective as assessment modality?	Yes [231,232,302-304]		No studies [304-306]		Emerging [231,232,304,305, 307,308]		Emerging [201,231,232, 302-305,307,308]		No studies [120,211,221,309-315]		Yes [231,252,312, 316-321]	
**Sleep-wake disorders**													
	RCT-TAU (patients/studies), n/n	—		—		1779/9		—		—		—	
	RTC-other (patients/studies), n/n	—		—		1220/13		—		—		—	
	Observational (patients/studies), n/n	—		—		334/6		—		—		—	
	Reported study quality (1=low to 3=high)	—		—		2.00 (SD 0.63)		—		—		—	
	Overall strength of evidence	—		—		Medium		—		—		—	
	Effective as treatment modality?	—		—		No		—		—		—	
	Effective as assessment modality?	—		—		No studies [182,322-328]		—		—		—	
**Substance use disorders**													
	RCT-TAU (patients/studies), n/n	8151/21		—		61,896/93		4650/8		11/1		—	
	RTC-other (patients/studies), n/n	15,610/31		—		97,802/180		12,385/22		219/5		—	
	Observational (patients/studies), n/n	984/5		—		14,603/35		5231/30		181/8		—	
	Reported study quality (1=low to 3=high)	2.13 (SD 0.74)		—		2.07 (SD 0.63)		2.26 (SD 0.74)		1.33 (SD 0.47)		—	
	Overall strength of evidence	High		—		High		High		Medium		—	
	Effective as treatment modality?	Yes		—		Yes		Yes		Inconclusive		—	
	Effective as assessment modality?	Yes [32,75,96-99, 329-345]		—		Yes [32,75,78, 96-114,188,277, 332-339,344,346-354]		Emerging [32,99,100, 333-339, 344,346-348,354-359]		No studies [115,120,211,221]		—	
**Total**													
	RCT-TAU (patients/studies), n/n	22,326/178		11/5		89,360/273		11,927/34		7909/140		3474/76	
	RTC-other (patients/studies), n/n	30,471/152		1018/13		163,655/569		22,521/63		8477/162		5494/125	
	Observational (patients/studies), n/n	10,465/252		210/5		48,802/166		7332/92		3718/152		796/63	
	Reported study quality (1=low to 3=high)	1.69		1.54		1.86		1.83		1.64		1.45	
	Overall strength of evidence	High		Low		High		Medium		High		Medium	
	Effective as treatment modality?	Yes		Emerging		Inconclusive		Inconclusive		Inconclusive		Yes	
	Effective as assessment modality?	Yes		No studies		Yes		Emerging		Emerging		Yes	

^a^RCT-TAU control: Randomized controlled trials with a treatment-as-usual control.

^b^—: not determined.

^c^RCT-other control: Randomized controlled trials with a waitlist or placebo control.

^d^High confidence based on >30 randomized controlled trials with >2000 participants in total; Medium confidence owing to <30 randomized controlled trials with <2000 participants; Low confidence owing to <500 participants; N/A: not applicable.

^e^Yes=positive treatment outcomes and low drop-out rates; Inconclusive=mixed findings, may be effective; Emerging=novel area of research with insufficient evidence; No=no significant difference in outcomes between intervention and controls.

^f^Web-based programs developed for bipolar disorders only address depression symptoms but not mania symptoms.

^g^Patients with schizophrenia and psychotic disorders or symptoms may not be willing to use any digital modalities owing to paranoia about technology, which stems from the underlying psychopathology.

Digital health interventions can be separated into 7 primary categories.

#### Synchronous and Asynchronous Therapist Contact

Synchronous contact refers to methods where providers and patients communicate at the same time (eg, phone call and videoconference). With better technology practitioners have gravitated toward videoconferencing, but a telephone call is used in the event of technical issues [232,360,361]. Delivery of assessment or treatment (eg, prescribing medication, parent and caregiver training, and various therapies) are usually provided using synchronous forms of communication. Nonetheless, it may also be more difficult to deliver time-dependent neurological tests [238]. Synchronous contact remains the primary form of treatment where other forms of treatment described below are only supplementary [68,69,84,98,109,111, 112,124, 135,151,159,169,170,178,179,199,273,348,349,362].

From a patient’s perspective, most felt that synchronous contact with a therapist afforded greater accessibility, independence, and made it easier for them to express themselves, others felt that it was impersonal [231,363], and some patients with schizophrenia and psychosis disorders were not comfortable with the technology, felt monitored or recorded, and refused care [147,232,308,360,361].

For asynchronous communications, there is a time delay between responses (eg, email and text). These methods can be useful in encouraging patients to attend their appointments, take their medications, exercise, relax, complete daily life tasks, and reduce relapse following remission [74,110,157,198,232,274,279,330,361,364-367]. However, asynchronous forms of communication were not as effective as synchronous forms of communication before remission [123,129,142,147,148]. Furthermore, asynchronous communications are rarely tested in emergency situations with patients who are potentially suicidal or violent [29,125,149,179].

#### Web-Based Peer Support

Mental health support provided by people with lived experience of mental health issues took place via web-based discussion groups (video calls, forums, and social media) where patients with similar disorders can interact. Treatment programs rarely include web-based groups, and few studies explore their role in treatment and adherence; therefore, we retained it as a separate category. Online communities and forums (eg, specific subreddits, forums, discord, and Facebook groups) are prevalent for all mental health conditions since patients can learn more about others’ experiences, learn about their condition, receive peer support, and accept their condition [87,232,304,305]. Online communities have also formed on YouTube where people living with mental health conditions are able to share their lived experience and insights. While there are examples of evidence-based forums and media content on mental health conditions, web-based content is not usually moderated, which may lead to the spread of misinformation. Indeed, unmoderated online communities have lower retention rates [306], suggesting that moderation by a practitioner may be required to reduce potential problems. Nevertheless, patient involvement and interaction on social media platforms provide significant insights, alternative perspectives, and fortitude to the general population, other patients, health care providers, and researchers. Despite their prominence, their use and effectiveness are rarely evaluated.

#### Web-Based or Computer-Based Therapy Programs

Various types of content delivered on the internet included psychoeducation, self-help therapy, journaling, assessments, topics traditionally covered in workbooks and paper format, reminders to take medication, motivational interventions, and web-based peer support. Web-based and mobile programs delivered with administrative or therapist guidance are as effective as treatment as usual (TAU), while those without guidance show significantly lower effectiveness and variable dropout rates [68,69,84,98,109,111,112,124,135, 151,159,169,170,178,179,199,273,308,348,349,362]. These are well developed for substance use–, mood-, anxiety-, and trauma-related disorders but not bipolar, personality, and sleep-wake disorders (Table 2). Indeed, for the latter disorders these interventions yield mixed results since they primarily treat anxiety and mood symptoms, but not mania or other symptoms [233,323,326].

#### Mobile-Based Therapy Programs

Mobile apps are a novel way to deliver therapy programs on mobile devices and share similarities to web-based or computer-based therapy programs. Over 2200 mobile apps claim to deliver therapy for several mental health conditions but lack rigorous validation, are not necessarily based on therapeutic principles, are gamified and addictive, or harm recovery [1,43,74,154,180,185,187,194-196,198,200,231,237,280,308,333,356,368]. Furthermore, 38% of trials for mobile apps were uncontrolled (Table 2). Mobile apps were therefore separated from web-based and computerized therapy (Table 2). We also urge caution when selecting mobile apps and provide a list of web-based tools and apps that have previously been validated (Table S1 in Multimedia Appendix 1).

#### Virtual and Augmented Reality

Virtual and augmented reality provide realistic and immersive experiences with a sense of presence for participants. It is a promising tool for new forms of assessment, treatment, and research to understand psychological processes (eg, psychosis and paranoia) [310]. Virtual reality is easier to implement, perform, and more realistic, motivating, and enjoyable than traditional exposure therapy [65-67,98,115,134,183,205,206,208-210,212-215, 219,222,311,369]. Virtual reality can be used to deliver psychotherapy, education, cognitive therapy, and exposure therapy [65-67,98,115,134, 183,205,206,208-210,212-215,217, 219,222,248,257,292,310,311,369,370]. Experiential cognitive therapy, a combination of virtual and cognitive therapy, has also been successful in treating eating- and weight-related disorders [284,371]. Lastly, virtual reality is valuable as a distraction tool, which leads to reduced pain perception, improved functional ability, and lower stress in patients with various acute and chronic illnesses [81,83,290,291,294,295]. Virtual reality could also provide otherwise inaccessible experiences to individuals with a disability, older individuals, or those living with a chronic illness or disability. Nevertheless, virtual reality should be part of a comprehensive treatment strategy [67].

Initial concerns that virtual reality could induce nausea, headaches, and other negative side effects, which could ultimately worsen phobias and attrition [207] have been assuaged by several improvements in the technology [67,206]. Practitioners should nevertheless use caution and test participants for susceptibility to motion sickness [218]. Some of these concerns may be addressed by using augmented reality where 3D representations of elements are imposed on the user’s native world, but more research is necessary for conclusive evidence of treatment efficacy between virtual reality and augmented reality [184]. Therapists also need to carefully assess for signs of cognitive avoidance in patients where they might treat virtual environment and stimuli as a “game” instead of cognitive immersion [218]. Mobile-based virtual reality treatments may provide new treatment avenues for patients who cannot attend in-person therapy owing to disability, transportation, or health concerns [137,220].

#### Cognitive Training

Cognitive training includes training exercises, neurofeedback, and games provided over mobile, web-based, or computer devices or virtual reality. These provide greater flexibility and development than pen-and-paper methods. Evidence suggests broad cognitive training is more effective than a narrow focus on a single cognitive modality [61,244,252,316,318]. Additionally, these must also be combined with tailored remediation to extract the greatest benefits in everyday life [58,92,244,252,258,316-318,372]. Cognitive declines are also reported in anxiety, mood, bipolar, and personality disorders, where similarly broad cognitive training could be useful to alleviate cognitive decline, reduce premature brain aging [59-62,64,150,251,252], and increase remission [166,230], and where the success of cognitive training in disorders such as schizophrenia, ADHD, developmental disorders, and dementia could be applied. Cognitive training and virtual reality could also improve broad motor and cognitive functions in patients with neurological disorders such as stroke, traumatic brain injury, Parkinson disease, and multiple sclerosis [61,247]. Attention bias modification appears to be successful in treating negative cognitive and attentional biases in patients with mood and anxiety disorders [163].

#### Other Technologies

Monitoring technologies (eg, breathalyzer, pill dispenser Wisepill, mobile apps, smart watches) are used to regularly monitor psychological symptoms, heart rate, blood pressure, location, and sleep and to alert practitioners to early signs of relapse, missed doses, or to flag early warning signs of disease [60,232,236,243,251,302,305,306,333,334]. Security systems, call screening technology (for scams), and chatbots can also improve quality of life, but more research is needed [60,243,245,251]. Lastly, transcranial direct current stimulation (tDCS) and similar treatments can be delivered remotely for dementia and schizophrenia [244].

## Discussion

### Principal Findings

This review found that a majority of studies on digital health interventions are focused on substance use–, anxiety-, mood-, and trauma-related disorders. For patients with these conditions, the greater flexibility, comfort, and routine associated with digital health offered a favorable substitute for in-person visits and retained therapeutic utility. Given this finding, we expect the use of digital health interventions to persist during and after the pandemic owing to the relaxation of insurance and administrative regulations [12,373-377]. The volume and quality of research for these disorders has enabled the discovery of new treatment methods and the refinement of existing digital health tools to improve treatment efficacy.

We also found that the sudden onset of the COVID-19 pandemic led to a rapid shift toward the use of new technology and interventions without the necessary time to train or prepare practitioners and posed challenges for many health care providers. To remedy this, governments, professional organizations, and academics, have created region-specific digital health toolkits [12,378-383] to facilitate and encourage the provision of digital health services. These toolkits are extensive and provide examples of ways in which digital health can be delivered in a meaningful and effective way.

Evidence from this review also suggests that digital health interventions have implications for combatting the dual public health emergencies across North America: the COVID-19 pandemic and the ongoing overdose crisis [11,384]. Findings indicate that there is significant potential for digital health interventions in reducing the harms experienced by people who use substances [32,75,78,96-114,329-339,346-353,385]. Research into digital health interventions for substance use disorders is relatively new and demonstrates the promising use of web-based programs and social media to reach participants instead of relying solely on referrals from practitioners [100,102,111-114,171,339,349,385]. These interventions may offer timely and cost-effective solutions, where texting, moderated forums, validated web-based or computer-based programs, or mobile apps may be used for treatment, psychoeducation, managing ongoing symptoms, and preventing relapse [12,20,25,30, 79,80,85,147,157,175,351] (see Table S1 in Multimedia Appendix 1 for a list of validated tools). Nevertheless, it is important to acknowledge that there are certain instances where in-person contact with a service provider is most suitable. This is particularly important given that many homeless and street-involved populations lack access to and knowledge of technology [386-388].

Similarly, this review found indications that web-based programs in anxiety-, mood-, and trauma-related disorders are poised for similar expansion. Since anxiety and depression symptoms have risen in the general population during the pandemic [10-16], several interventions can be useful for short-term symptom management, such as synchronous communication (videoconferencing or telephone calls) with a therapist [68,96,123-126,129,130,133-139,141,142] and referral to validated web-based [1,30,68,69,84-86,96,118,124, 125,129,130,133-135,137,138,142,145-187], computer-, or mobile-based applications [1,68,96,185-187,193-200] such as those listed in Table S1 in Multimedia Appendix 1. However, we would like to emphasize that interventions were far less successful without practitioner guidance [30,69,73,76,77,155,157,252].

While this review also identified promising developments in digital programs for ADHD, developmental, dementia, eating, schizophrenia, and chronic illness, we found that digital health interventions for these conditions are nascent. Negative findings in sleep-wake and bipolar disorders suggest that significant retooling is necessary for treating these conditions. Furthermore, no reviews on the use of digital health tools for dissociative, elimination, sexual, and personality disorders were identified. The positive outcomes reported for digital health interventions in a wide range of mental health conditions suggest that there may be merit to exploring these interventions in additional clinical contexts during and after the COVID-19 pandemic. Caution is also warranted with patients with schizophrenia, psychosis, or bipolar disorder as technology may be triggering or could exacerbate existing symptoms [147,232,360,361].

Review findings also suggest that synchronous digital contact is an effective substitution for in-person treatment and assessment for many mental health conditions. Considering successes in most mental health conditions, these findings can be generalized to other conditions where less research is available, such as bipolar, sleep-wake–related, and personality disorders. While some health care providers have expressed concerns regarding their ability to build a therapeutic alliance with their patients, research shows that this is not significantly affected by synchronous communication [26,389,390]. Interestingly, synchronous digital health may be beneficial for autism spectrum disorders [269] and social anxiety since it reduces social interaction–related stress, need for eye contact, oversensitivity, and overstimulation. Evidence from this review indicates that synchronous platforms are associated with significant cost and time savings. First, this transition is also beneficial by reducing commutes to work, the ability to organize one’s working day and tasks [391-393], and protects therapists from the risk of physical confrontations [394,395].

Digital health tools have also been found to allow practitioners to reduce the time they spend with each patient, where evidence suggests that spending 10 minutes with patients through synchronous platforms, and providing referrals to asynchronous platforms (eg, web-based, mobile-, or computer-based therapy and cognitive training) is sufficient [30,69,73,76,77,155,157,252,308]. Some patients (eg, children and elderly) may face other barriers to using or accessing technology [396,397], which can be resolved by specific training on using the application [59,62], obtaining help from a caregiver, and could even be accomplished through remote desktop applications (such as Microsoft Teams: Remote Desktop Protocol). Nonetheless, transferring this responsibility to a family member increases caregiver burden and may lead to suboptimal results over the long term. However, the proliferation of untested applications (especially mobile apps) raises concerns around the quality of existing platforms [1,74,154,180,185,187,194-196,198,200,231,237,280,333]. More specifically, these applications often lack validation, reliability, and are not always built on sound psychotherapeutic principles [1,74,154,180,185,187, 194-196,198,200,231,237,280,308,333].

Digital health interventions are also less effective at mitigating the impacts of social isolation, particularly in the context of the COVID-19 pandemic, where public health orders and the requirement of physical distancing is expected to drastically impact peoples’ mental health. Human connection contributes significantly to one’s mental health; therefore, it is important that digital health interventions maintain their human aspect as this is associated with increased efficacy [68,69,84,98,109,111,112, 124,135,151,159,169,170,178,179,199,273,348,349,362]. Findings demonstrate that asynchronous platforms, such as web-based forums, social media, and other digital communities, likely increase patient engagement and adherence to treatment across all mental health conditions [87,232,304,305]. Additionally, preventative education can be disseminated via asynchronous platforms (eg, social media, groups, forums, and schools) for all mental health conditions, as seen in substance use disorders [100,102,111-114,171,339,349]. Owing to increased demand and lack of availability of services during the COVID-19 pandemic, many patients have transitioned to mobile apps and web-based programs without the guidance of a practitioner [13]. Hence, the absence of sufficient research into these venues, their impact on mental health, and the lack of practitioner guidance and support [1,74,154,180,185,187,194-196,198,200,231,237,280,333] raise concerns that these platforms may cause harm. Indeed, government intervention to increase the prominence of validated region-specific tools and resources in web-based and app-related searches may be required.

Another emerging asynchronous technology that can be used for the treatment of mental health conditions are virtual reality tools. Greater accessibility, comfort, and normalcy of the technology will encourage the development of virtual reality interventions on site or at home. Nevertheless, there are also barriers to providing and expanding virtual reality tools. For example, the high cost of equipment acts as a significant barrier, however, lower priced equipment or mobile phones can be used as substitutes [137,220,247]. Additionally, virtual reality tools are based on recent technological advancements, and there is little quality research on the use of industry-standard equipment and even less so for low-cost virtual reality options. Despite these limitations, virtual reality addresses a particular niche of therapeutic tools (eg, exposure therapy) [65-67,98,115,134,183,205,206,208-210,212-215,219,222,311,369] and is an effective tool for pain management [81,83,290,291,294,295], indicating that as technology and research advances, it may become a central component of any comprehensive mental health treatment strategy.

Owing to the social distancing and quarantine requirements posed by the pandemic, patients with mental health disorders already face social isolation in addition to increased stress and anxiety [5-14]. Additionally, patients surviving COVID-19 may experience lingering symptoms and post–intensive care syndrome long after discharge from intensive care units [398,399]. Mental health challenges for these patients include anxiety, depression, posttraumatic stress disorder (PTSD), cognitive decline, and chronic illness [398,399]. Along with previously mentioned interventions to deal with symptoms of anxiety, depression, and posttraumatic stress, virtual reality can be used to reduce stress, distract from pain, and retrain functional movement in patients who experience chronic illness after COVID-19.

Health care providers are also at risk of feeling social and professional isolation as well as burnout [26,394,400], which must be properly managed by managers, the professional organization, and practitioners themselves. Given the anticipated impact of the pandemic on the mental health of health care providers [11,18,401-403], health care organizations will benefit from specialized synchronous, web-based, and mobile therapy and moderated discussion forums to alleviate this burden. Similar interventions have been used with family caregivers [70,88-90] and health care providers [18,401,402,404,405] to treat anxiety, depression, PTSD, and burn out. Therefore, such interventions can help manage health care providers’ mental health.

### Future Directions

Over the last two decades, research on the use of digital health interventions to deliver mental health care has increased significantly. Lessons learned from highly studied fields (eg, substance use–, anxiety-, mood-, and trauma-related disorders) can guide the implementation of digital health interventions to treat other mental health conditions. Starting at the most basic level, where practitioner guidance for 10 minutes was essential and often sufficient for the treatment of anxiety-, mood-, and trauma-related disorders, web-based, computer-based, or mobile programs or apps developed for these conditions could be adapted, improved upon, and evaluated to treat other conditions with overlapping symptomatology. For example, one could consider the overlap in symptomatology among mood-related, anxiety, bipolar, sleep-wake–related, and some personality disorders [406]. Thus, digital interventions for the former two conditions could be adapted to include journaling, behavioral modification prompts, and other psychotherapeutic treatments akin to these conditions, and finally be re-evaluated. Nonetheless, for conditions where no treatments exist, the development and digitization of novel treatment strategies is required [233,323,326]. Indeed, the digital nature of these programs enables the collection of regular assessment data, input from patients, and evaluation by health care providers to develop decision trees and machine learning algorithms to instantly improve and personalize treatment plans, require less practitioner time, and provide greater flexibility in treatment delivery.

The rapid pace of technological advancements also poses significant challenges. For example, treatment program implementation has evolved from computerized delivery with CDs to web platforms to mobile apps in the last two decades. Significant technological shifts have forced researchers to completely rebuild the programs despite apparent similarities between these modes of delivery. Many validated programs identified (Table S2 in Multimedia Appendix 1) are outpaced by technological advancements and lack recent updates. First, easy-to-use development and cross-platform tools (eg, React Native and Xamarin) will enable researchers to make, evaluate, and maintain programs despite rapid technological advancements. Second, health care policies and evaluation may need to be modified so that validated tools can evolve over time and across platforms when the underlying therapeutic principles remain consistent.

Existing research on digital interventions rarely covers comorbid conditions, emergency situations, or complex socioeconomic factors. For example, research on people experiencing homelessness is limited to commentaries and policy recommendations based on available research in the general population [32-42]. This is of particular concern when considering that those of lower socioeconomic status or with complex life circumstances show reduced benefits from digital health interventions [148,165,407]. Additional research considering individuals experiencing various psychosocial complexities or comorbid conditions is required.

Further research must endeavor to use appropriate controls and more rigorous design to improve overall study quality assessed in Table 2. Blinding patients to the digital nature of the treatment is difficult, but creative solutions (eg, unrelated cognitive tasks in lieu of treatment) are recommended. Additionally, standardized rating scales (ie, DSM-V criteria) should be used instead of nonstandard assessments or a participant’s opinion on the treatment. Most studies are restricted to treatment duration and lack long-term follow-up (>6 months). Considering digitization of treatments and records, practitioners can automatically request follow-up surveys and assessments via email or text. Follow-up surveys must also consider whether patients have pursued other treatment programs, as these could confound any pertinent treatment effects. Lastly, following successful remission, there is limited research on the use of digital health interventions (eg, email, text, social media, and forums) to prevent relapse, which can be accomplished via email or text [74,110,157,198,232,274,279,330,361,364-367].

Web-based peer support is dependent on human interaction, which can be unpredictable and include uncontrolled variables. For example, since any large number of people can participate in forums for intermittent periods of time, the inevitable turnover can cause cultural shifts. This would therefore require moderation by practitioners. Evaluation is further complicated by the lack of objective and quantifiable pre-post measures in open social media groups and forums. Indeed, practitioner moderated forums or groups may fare better and could automatically request participants to fill out monthly surveys. Further research is needed to address these hypotheses.

### Limitations

There are several limitations to this review. First, to rapidly inform health care stakeholders responsible for managing treatment of a broad range of mental health disorders, we took a comprehensive approach. As a result, we limited the scope to secondary literature sources and utilized a systematic methodology designed for meta-reviews [167]. Since we primarily report on the effectiveness, feasibility, and reliability of digital delivery in lieu of face-to-face treatment, we did not attempt to compare different forms of therapy. This included drawing comparisons to nondigital interventions reported within identified studies, when available (27% of primary studies compared digital health interventions to TAU). Owing to the urgency of this endeavor and to limit the already substantial number of references, we focus exclusively on reports obtained from Medline. This is not atypical as many of the included reports use a single database but can miss some reports.

Additionally, the metadata collection procedure described only approximates the state and volume of research. Reliance on secondary research articles implies that we likely missed recent relevant primary research articles. Nevertheless, our correlative analysis (Table S2 and Figure S1 in Multimedia Appendix 1) suggests that metadata and secondary research can be used to estimate the relative amount and reliability of primary research.

Owing to differences in quality and bias reporting between included literature, we could not report this for individual studies and instead relied on the included literature sources to dictate the quality of research in the field. We observed considerable variability in quality assessments between reviews (Table 2). Potential explanations include the specific selection and inclusion or exclusion criteria of reviews or lower stringency in early discovery studies versus later RCTs. Nevertheless, this raises concerns regarding interreview reliability, which we did not assess here. To enable policy makers and researchers to reliably compile all amassed data, reliably rate studies, and reduce time lost to re-evaluating studies we recommend an update to Cochrane and PRISMA requirements to include the adoption of a single consistent bias and quality assessment reporting methodology and consistent reporting of study details in all reviews. In addition to ensuring similar quality and bias assessment between reviewers within a review, we recommend comparison with previous reviews to ensure greater reproducibility of quality and bias assessments between independent reviews. Nevertheless, living systematic reviews are likely to accelerate research and development in digital mental health interventions and may if designed accordingly upend the systematic review process. Living reviews stem from the ability to continuously update web-based articles with the latest developments in the field. These are a way forward for rapid evidence-based development, collaboration, standardization of digital health tools, and a necessary step forward to improve treatment options.

### Conclusions

Although digital delivery of mental health treatment has been in clinical use for a long time, the available research on the topic is far from comprehensive or consistent. New guidelines to increase reliability and consistency of reporting, evaluation, and quality and bias assessments would enable faster literature synthesis and increase confidence. Living systematic reviews for bipolar, personality, developmental, dementia, and sleep-wake disorders would also be very useful to guide and organize novel digital treatment strategies.

Overall, digital treatment strategies paired with synchronous practitioner contact are as effective as nondigital alternatives. However, in offering digital treatments, it is essential to consider feasibility of treatment, caregiver burden, patient-specific symptoms (eg, paranoia), and patient-specific parameters. More research is especially needed in marginalized populations who face greater barriers to mental health treatment access. Thus, to maintain treatment quality and efficacy, patients should have the option for face-to-face interventions, despite the challenges posed by the COVID-19 pandemic. Nonetheless, digital treatments offer many benefits such as increased patient engagement, accessibility, and availability paired with reduced practitioner workload. Additionally, the drastic shift to digital health is likely to encourage further developments in treatments for many mental health disorders and expansion into other digital modalities, such as virtual reality, social media, and web-based forums. These developments promise significant advances in mental health treatment via global collaboration and investment.

## Appendix

Multimedia Appendix 1 Supplementary.

Multimedia Appendix 2 Extracted data for digital health interventions per mental health condition.

## Abbreviations

ADHD: attention-deficit/hyperactivity disorder
DSM-5: Diagnostic and Statistical Manual of Mental Disorders, Fifth Edition
MeSH: Medical Subject Headings
PRISMA: Preferred Reporting Items for Systematic Reviews and Meta-Analyses
PTSD: post-traumatic stress disorder
TAU: treatment as usual
