# Supplementary material for: Digital Health Interventions for Delivery of Mental Health Care: Systematic and Comprehensive Meta-Review
Source: JMIR Ment Health. 2022 May 12;9(5):e35159. doi: 10.2196/35159 (PMC9109782; doi:10.2196/35159)
Supplement: Multimedia Appendix 1 [file mental_v9i5e35159_app1.pdf]

Appendix

Table of contents

Page	Content
1	Appendix contents
2	Figure S1
3-4	Table S1
5	Table S2
6-7	Prisma checklist
8	Search strategy
Other	Excel spreadsheet containing raw extracted data

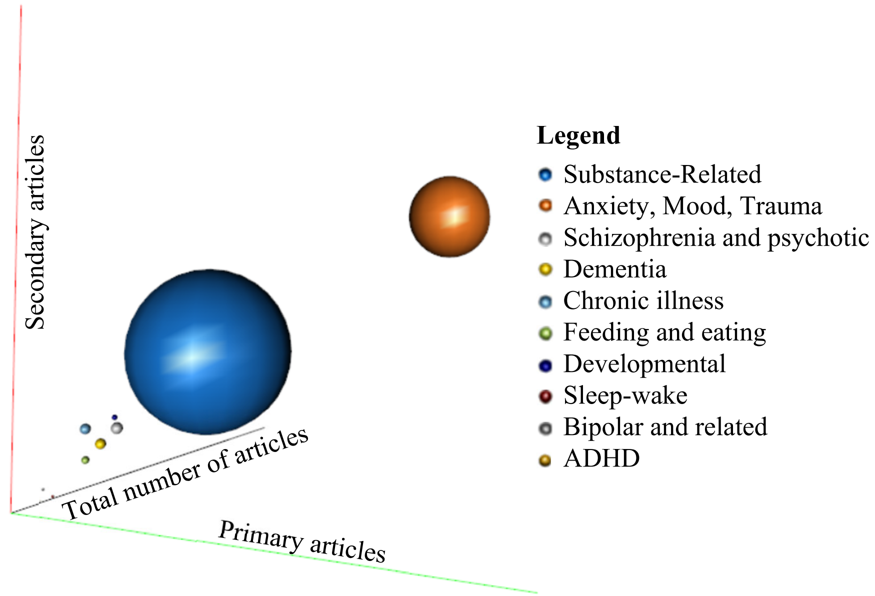

Figure S1. Four-dimensional representation of the total literature (X axis, black), **curated secondary literature** (Y axis, red)**, primary literature curated elsewhere** (Z axis, green) **and participant number** (sphere size)**.** For graphical purposes these were collapsed across digital health intervention and expressed as percentages. Interactive graphic available here: https://goofy-fermat-ef5a3a.netlify.app/.

Table S1. Websites, programs, apps, social media and forums evaluated elsewhere per mental health condition.

Area	Resource
Technological transition	● DOORS program: https://www.digitalpsych.org<br>● Doctors of BC toolkits: https://www.doctorsofbc.ca/managing-your-practice/doctors-technology-office-dto/virtual-care<br>● Canadian Medical Association tookits: https://www.cma.ca/virtual-care<br>● General guide: https://www.sciencedirect.com/science/article/pii/S1056499319300288?via%3Dihub
Attention deficit hyperactivity disorder	● Apps: ADHD trainer<br>● Computer games: Costum-made, Rayman Raving Rabbids, Calcularis, Adventurous dreaming highflying dragon, Brain game Brian.<br>● Plan-It commander: www.planitcommander.com<br>● CogMed: www.cogmed.com
Anxiety, Trauma, Stress, Mood	● Apps: Fear Fighter, PTSD Coach, SuperBetter, ibobbly, ACTsmart, CBM Active,<br>● EVO, Get Happy, Headspace, LivingSmart, Mission Reconnect, MobileType, MONARCA, MoodHacker, My Compass, PRISM, SMART, for a comprehensive review of mobile apps see [79].<br>● Beating the blues: www.beatingtheblues.co.uk<br>● Brave online: https://www.brave-online.com/<br>● BTsteps: https://waypointhealth.com/bt-steps/<br>● Cool Teens: https://www.c4tbh.org/program-review/cool-teens/<br>● Good days ahead: www.empower-interactive.com/solutions/good-days-ahead/<br>● Mood gym: https://moodgym.com.au/<br>● myMCT: https://clinical-neuropsychology.de/metacognitive-training-for-ocd-mymct/ODIN [333]<br>● The Sadness program: https://www.c4tbh.org/program-review/the-sadness-program/<br>● The Shyness program: https://thiswayup.org.au/how-we-can-help/courses/social-phobia/<br>● STRIVE: http://www.strivebhc.com/<br>● VetChange: https://vetchange.org<br>● Worry Program: https://thiswayup.org.au/how-we-can-help/courses/generalised-anxiety-disorder/<br>● Emotional Regulation program: GameTeen – computer and mobile [20]
Bipolar disorders	● Apps: Health buddy<br>• Beating Bipolar: http://beatingbipolar.org/<br>• Recovery road: https://recoveryroadmc.com/
Dementia	• CogPack: http://www.cogpack.com/USA/info.htm
Dementia - Caregiver support	• understAID: http://www.aal-europe.eu/projects/understaid/<br>• ALADDIN: http://www.aal-europe.eu/projects/alladin/
Developmental disorders (exclude ADHD)	• TeachTown: http://web.teachtown.com/<br>• Fast ForWord: https://www.scilearn.com/program/<br>• Mencap's Family Hub: www.mencap.org.uk/familyhub<br>• Computer games: Second Live<br>• ImPACT<br>• Forums: https://www.autismforums.com/ [1]<br>• Aspergers from the Inside: https://aspergersfromtheinside.com/ and https://www.youtube.com/channel/UC-FpBZR7DbpvNj5UrFN8qUA [1]<br>• Yo Samdy Sam: https://yosamdysam.com/ and https://www.youtube.com/user/AtheistRockstar [1]
Feeding and eating disorders	• Apps: RR eating disorder management; Rise up: eating disorder help; Psychiatry prodiagnosis, info, treatment, CBT and DBT [1]<br>• Online programs: My body[334], Feedback

	• Salut BED: https://www.netunion.com/salut_bed/<br>• Salut BN: https://www.netunion.com/salut_bn/index.php<br>• Overcoming Bulimia Online: https://overcomingbulimia.com/<br>• BIVRS – Body Image Virtual Reality Scale software [297]<br>• VEBIM – Virtual Environment for Body Image Modification as part of NeuroVR www.neuroVR.org [297]
Schizophrenia and psychotic disorders	• Apps: PRIME, CLIMB, BiasBlaster, Sleepio, PsyMate, MindFrame, AiCure, App4Independence, Actissist, and BrainHQ<br>• HORYZONS: www.ymhtech.com/horyzonscanada<br>• Cogpack: http://www.cogpack.com/USA/info.htm<br>• Cogrehab: https://sites.google.com/site/cogrehab/<br>• Living well with Schizophrenia: https://www.livingwellwithschizophrenia.org and https://www.youtube.com/channel/UCENqBB_xNax3mLX_WGLf2Lg [1]
Sleep-wake disorders	• App: Sleepcare, SHUTi, Sleepio, GET.ON
Substance- related disorders	• Apps: A-CHESS, Promillekoll, LBMI-A, QuitGuide, SmartQuit, Craving to Quit, Bounce Back Now!<br>• SHADE: www.c4tbh.org/program-review/shade-self-help-for-alcohol-and-other-drug-use-and-depression/<br>• DEAL [335]<br>• SaferTeens: www.saferteens.org/home/<br>• VetChange: https://vetchange.org<br>• SmokeFree: www.smokefree.gov<br>• AlcoholScreening: www.alcoholscreening.org<br>• Becomeanex: www.becomeanex.org<br>• CHOICES: www.njchoices.org<br>• Rx for Change: rxforchange.ucsf.edu<br>• Freedom from Smoking Online: www.ffsonline.org<br>• ClimateSchools: www.climateschools.com<br>• Forums: www.soberrecovery.com, https://www.facebook.com/LeaveThePackBehind/<br>• Self and impulse control: PlayMancer – VR game[20]

1. Resources not evaluated by previous studies but deserving of mention and further inquiry.

Self and impulse control: PlayMancer – VR game [20]
Resources not evaluated by previous studies but deserving of mention and further inquiry.

Table S2. Correlational relationship between meta-data analysis across mental health conditions.

	**Total literature**	**Secondary literature**	**Primary literature**	**Number of participants**
**Total articles**	1	$r^2 = .988$<br>$p > 0.000$	$r^2 = .944$<br>$p > 0.000$	$r^2 = .685$<br>$p = 0.029$
**Secondary articles**	$r^2 = .988$<br>$p > 0.000$	1	$r^2 = .953$<br>$p > 0.000$	$r^2 = .707$<br>$p = 0.022$
**Primary articles**	$r^2 = .944$<br>$p > 0.000$	$r^2 = .953$<br>$p > 0.000$	1	$r^2 = .666$<br>$p = 0.035$
**Number of participants**	$r^2 = .685$<br>$p = 0.029$	$r^2 = .707$<br>$p = 0.022$	$r^2 = .691$<br>$p = 0.027$	1

Prisma checklist

Section/topic	#	Checklist item	Reported on page #
**TITLE**			
Title	1	Identify the report as a systematic review, meta-analysis, or both.	1
**ABSTRACT**			
Structured summary	2	Provide a structured summary including, as applicable: background; objectives; data sources; study eligibility criteria, participants, and interventions; study appraisal and synthesis methods; results; limitations; conclusions and implications of key findings; systematic review registration number.	2
**INTRODUCTION**			
Rationale	3	Describe the rationale for the review in the context of what is already known.	3-5
Objectives	4	Provide an explicit statement of questions being addressed with reference to participants, interventions, comparisons, outcomes, and study design (PICOS).	4-5
**METHODS**			
Protocol and registration	5	Indicate if a review protocol exists, if and where it can be accessed (e.g., Web address), and, if available, provide registration information including registration number.	NA
Eligibility criteria	6	Specify study characteristics (e.g., PICOS, length of follow-up) and report characteristics (e.g., years considered, language, publication status) used as criteria for eligibility, giving rationale.	5-6
Information sources	7	Describe all information sources (e.g., databases with dates of coverage, contact with study authors to identify additional studies) in the search and date last searched.	5
Search	8	Present full electronic search strategy for at least one database, including any limits used, such that it could be repeated.	Appendix 8
Study selection	9	State the process for selecting studies (i.e., screening, eligibility, included in systematic review, and, if applicable, included in the meta-analysis).	5-6
Data collection process	10	Describe method of data extraction from reports (e.g., piloted forms, independently, in duplicate) and any processes for obtaining and confirming data from investigators.	6
Data items	11	List and define all variables for which data were sought (e.g., PICOS, funding sources) and any assumptions and simplifications made.	6
Risk of bias in individual studies	12	Describe methods used for assessing risk of bias of individual studies (including specification of whether this was done at the study or outcome level), and how this information is to be used in any data synthesis.	6
Summary measures	13	State the principal summary measures (e.g., risk ratio, difference in means).	NA
Synthesis of results	14	Describe the methods of handling data and combining results of studies, if done, including measures of consistency (e.g., I^2) for each meta-analysis.	6

Risk of bias across studies	15	Specify any assessment of risk of bias that may affect the cumulative evidence (e.g., publication bias, selective reporting within studies).	6
Additional analyses	16	Describe methods of additional analyses (e.g., sensitivity or subgroup analyses, meta-regression), if done, indicating which were pre-specified.	8-9<br>Appendix 2, 5
**RESULTS**			
Study selection	17	Give numbers of studies screened, assessed for eligibility, and included in the review, with reasons for exclusions at each stage, ideally with a flow diagram.	8
Study characteristics	18	For each study, present characteristics for which data were extracted (e.g., study size, PICOS, follow-up period) and provide the citations.	Supplementary excel file
Risk of bias within studies	19	Present data on risk of bias of each study and, if available, any outcome level assessment (see item 12).	Supplementary excel file
Results of individual studies	20	For all outcomes considered (benefits or harms), present, for each study: (a) simple summary data for each intervention group (b) effect estimates and confidence intervals, ideally with a forest plot.	Supplementary excel file
Synthesis of results	21	Present results of each meta-analysis done, including confidence intervals and measures of consistency.	8-14
Risk of bias across studies	22	Present results of any assessment of risk of bias across studies (see Item 15).	67-68, Supplementary excel file
Additional analysis	23	Give results of additional analyses, if done (e.g., sensitivity or subgroup analyses, meta-regression [see Item 16]).	8-9<br>Appendix 2, 5
**DISCUSSION**			
Summary of evidence	24	Summarize the main findings including the strength of evidence for each main outcome; consider their relevance to key groups (e.g., healthcare providers, users, and policy makers).	14-20
Limitations	25	Discuss limitations at study and outcome level (e.g., risk of bias), and at review-level (e.g., incomplete retrieval of identified research, reporting bias).	22-24
Conclusions	26	Provide a general interpretation of the results in the context of other evidence, and implications for future research.	20-22<br>24-25
**FUNDING**			
Funding	27	Describe sources of funding for the systematic review and other support (e.g., supply of data); role of funders for the systematic review.	None

From: Moher D, Liberati A, Tetzlaff J, Altman DG, The PRISMA Group (2009). Preferred Reporting Items for Systematic Reviews and Meta-Analyses: The PRISMA Statement. PLoS Med 6(7): e1000097. doi:10.1371/journal.pmed1000097

Search query
("Virtual Reality Exposure Therapy"[MeSH] OR "telepsych*"[tiab] OR "telemental"[tiab] OR "teletherapy"[tiab] OR "virtual therapy"[tiab] OR "virtual mental"[tiab] OR "virtual psych*"[tiab]) OR (("Therapy, Computer-Assisted"[Majr] OR "Telemedicine"[Majr] OR "Videoconferencing"[Majr] OR "Remote Consultation"[MeSH] OR "Mobile Applications"[MeSH]) AND ("Mental Health Services"[Majr] OR "Mental disorders/therapy"[MeSH] OR "Psychotherapy"[Majr])) AND (“Attention Deficit and Disruptive Behavior Disorders”[MeSH] OR “Anxiety disorders”[MeSH] OR “Trauma and stressor related disorders”[MeSH] OR “Mood disorders”[MeSH] OR “Bipolar and related disorders”[MeSH] OR “Dementia”[MeSH] OR “Disruptive, impulse control and conduct disorders”[MeSH] OR “Dissociative disorders”[MeSH] OR “Elimination disorders”[MeSH] OR “Feeding and eating disorders”[MeSH] OR “Neurodevelopmental disorders”[MeSH] OR “Neurotic disorders”[MeSH] OR “Pain”[MeSH] OR “Paraphilic disorders”[MeSH] OR “Personality disorders”[MeSH] OR “Schizophrenia spectrum and other psychotic disorders”[MeSH] OR “Sexual dysfunction, psychological”[MeSH] OR “Sleep wake disorders”[MeSH] OR “Substance-related disorders”[MeSH])
